# Endotheliopathy in Acute COVID-19 and Long COVID

**DOI:** 10.3390/ijms24098237

**Published:** 2023-05-04

**Authors:** Alice G. Vassiliou, Charikleia S. Vrettou, Chrysi Keskinidou, Ioanna Dimopoulou, Anastasia Kotanidou, Stylianos E. Orfanos

**Affiliations:** First Department of Critical Care Medicine & Pulmonary Services, School of Medicine, National and Kapodistrian University of Athens, Evangelismos Hospital, 106 76 Athens, Greece; vrettou@hotmail.com (C.S.V.); chrysakes29@gmail.com (C.K.); idimo@otenet.gr (I.D.); akotanid@med.ua.gr (A.K.)

**Keywords:** endotheliopathy, biomarkers, COVID-19, long COVID

## Abstract

The pulmonary endothelium is a highly regulated organ that performs a wide range of functions under physiological and pathological conditions. Since endothelial dysfunction has been demonstrated to play a direct role in sepsis and acute respiratory distress syndrome, its role in COVID-19 has also been extensively investigated. Indeed, apart from the COVID-19-associated coagulopathy biomarkers, new biomarkers were recognised early during the pandemic, including markers of endothelial cell activation or injury. We systematically searched the literature up to 10 March 2023 for studies examining the association between acute and long COVID-19 severity and outcomes and endothelial biomarkers.

## 1. Introduction

Due to the progressive nature of coronavirus disease 19 (COVID-19), numerous predictor models have been developed using risk factors, comorbidities, epidemiological data, and basic laboratory values. Unfortunately, none of the aforementioned combinations can offer a precise and early prediction, due to the disease’s variability. Moreover, it has been proven very difficult to recognise the clinical features of COVID-19 and to identify appropriate therapeutic options due to the viral mutations affecting clinical response and epidemiological indicators [1,2,3]. Hence, adding characteristics that are more relevant to the pathobiology of the disease could significantly improve the prognostic value. Endothelial biomarkers are promising indices in outcome prediction since endothelial damage occurs in the early stages of many vascular illnesses. The pulmonary endothelium is a highly regulated organ that performs a wide range of functions under physiological and pathological conditions. Since endothelial dysfunction has been demonstrated to play a direct role in sepsis and acute respiratory distress syndrome (ARDS) [4], its role in COVID-19 has also been extensively investigated. Indeed, apart from the COVID-19-associated coagulopathy biomarkers, new biomarkers were recognised early during the pandemic, including markers of endothelial cell activation or injury [5].

As the pandemic has gradually been controlled, attention is now being shifted towards the long-term effects of COVID-19 on the health of survivors. Studies are being conducted to investigate the role of endothelial dysfunction in the long COVID syndrome. Long COVID is a condition that occurs in individuals who have had a confirmed or probable severe acute respiratory syndrome coronavirus 2 (SARS-CoV-2) infection, with symptoms lasting for at least two months that cannot be explained by any other diagnosis. The symptoms of long COVID include fatigue, shortness of breath, and cognitive dysfunction, which can impact daily life [6]. These symptoms can either be newly developed following the initial recovery from COVID-19 or persist from the initial illness. They are sometimes similar to those seen in encephalomyelitis/chronic fatigue syndromes (EM/CFS) that have been observed after infections with various viruses, including other coronaviruses [7].

In an effort to identify the extent of endothelial dysfunction in acute and long COVID-19, we systematically searched the literature (Pubmed database) up to March 10, 2023 for studies examining the association between acute and long COVID-19 severity and outcomes and endothelial biomarkers, including vascular endothelial (VE)-cadherin, syndecan-1, endocan, angiopoietin-1 and 2 (Ang-1, Ang-2), soluble (s)E-selectin, sP-selectin, soluble intercellular adhesion molecule 1 (sICAM-1), soluble vascular cell adhesion molecule 1 (sVCAM-1), soluble platelet endothelial cell adhesion molecule 1 (sPECAM-1), vascular endothelial growth factor (VEGF), ephrin-A1 and ephrin type-A receptor 2, soluble triggering receptor expressed on myeloid cells 1 (sTREM-1), CD40 ligand (CD40L), the soluble form of the urokinase plasminogen activator (suPAR), presepsin, von Willebrand factor (vWf), ADAMTS-13, tissue factor (TF), soluble endothelial protein C receptor (sEPCR), soluble thrombomodulin (sTM), plasminogen, plasminogen activator inhibitor 1 (PAI-1), endothelin-1, soluble angiotensin converting enzyme (sACE), soluble angiotensin converting enzyme 2 (sACE2), endothelial nitric oxide synthase (eNOS), and adrenomedullin (ADM). The rapidly emerging data into how endothelial dysfunction may contribute to the pandemic may lead to the identification of prognostic biomarkers, as well as treatments targeting pathogenic endothelial responses.

## 2. Endothelial Functions

**Figure 1:**
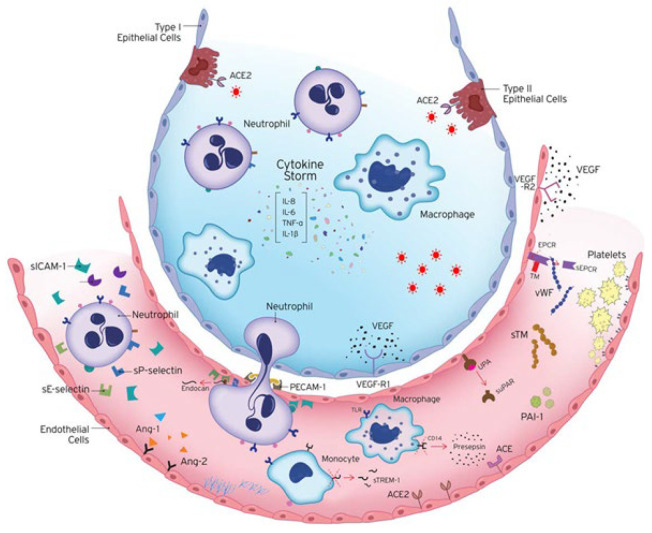
Endotheliopathy in COVID-19. COVID-19 is characterised by extended endothelial dysfunction. The host immune response to SARS-CoV-2 infection includes the excessive activation of immune cells, releasing vast amounts of pro- and anti-inflammatory cytokines, such as IL-1β, IL-6, IL-8, and TNF-α. Endothelial cells are activated and express adhesion molecules, to which immune cells bind, affecting coagulation, endothelial activation/dysfunction, and vascular barrier permeability. Ang-1 maintains vascular integrity, while Ang-2 counteracts the protective effects of Ang-1. Hence, the release of Ang-2 directly reflects vascular barrier breakdown. The endothelium becomes leaky and inflamed, allowing the transmigration of immune cells to the site of injury. A crucial regulator of endothelial cell homeostasis, tissue oedema, and inflammatory processes is the endothelial glycocalyx (EG). Microvascular leakage can result from glycocalyx dysfunction. Prior to their strong adherence and diapedesis at sites of infection and inflammation, active neutrophils adhere to endothelia through E-selectin and P-selectin. Their strong adherence to the endothelium is controlled by VCAM-1 and ICAM-1, and their subsequent transendothelial migration to infection sites occurs via PECAM-1. In response to pro-inflammatory cytokines and pro-angiogenic substances, the vascular endothelium expresses and secretes endocan, which prevents leukocyte migration. The soluble form of uPAR, suPAR, reflects general activation of the immune system. sTREM-1 is released during infection. Presepsin is an emerging biomarker of infection released by the cleavage of CD14. Coagulation and fibrinolysis are also critical host responses to infection by SARS-CoV-2. The anti-thrombotic, anti-inflammatory, and pro-fibrinolytic phenotype of endothelial cells (ECs) changes to an active state of endothelial dysfunction. ECs actively control haemostasis through the production of pro-thrombotic substances (von Willebrand factor, P-selectin), molecules that restrict coagulation (thrombomodulin), and fibrinolytic factors (plasminogen activators). Up-regulation of the plasminogen activator inhibitor-1 (PAI-1), the major inhibitor of fibrinolysis, leads to a shift from pro- to anti-fibrinolytic phenotypes. The protein C anticoagulant system also involves protein S, and the endothelial receptors TM and EPCR. A soluble form of EPCR (sEPCR) exists in conditions marked by enhanced inflammation. The vascular endothelium also maintains the vascular tone by producing vasoconstrictors and vasodilators, including endothelin-1, angiotensin-2, nitric oxide, and prostacyclin. ACE hydrolyses angiotensin I to angiotensin II and the balance between ACE and ACE2 is thought to be the key regulator of angiotensin II levels. Studies have implicated vascular endothelial growth factors (VEGFs), angiopoietins, and ephrins as key players in vascular development. VEGF is a glycoprotein originally isolated as a permeability factor with unique specificity for vascular ECs but is now known for its mitogenic and angiogenic properties. VEGF and its receptors VEGF-R1 and VEGF-R2 regulate angiogenesis and vascular permeability. ACE, Angiotensin converting enzyme; Ang-1, angiopoietin-1; Ang-2, angiopoietin-2; CD14, cluster of differentiation 14; EC, endothelial cell; IL, interleukin; PAI-1, plasminogen activator inhibitor 1; sEPCR, soluble endothelial protein C receptor; sICAM-1, soluble intercellular adhesion molecule 1; sPECAM-1, soluble platelet endothelial cell adhesion molecule 1; sTM, soluble thrombomodulin; sTREM-1, soluble triggering receptor expressed on myeloid cells 1; suPAR, soluble urokinase-type plasminogen activator receptor; sVCAM-1, soluble vascular adhesion molecule 1; TNF-α, tumour necrosis factor alpha; UPA, urokinase-type plasminogen activator; VEGF, vascular endothelial growth factor; VEGF-R, VEGF receptor; vWf, von Willebrand factor. The image is adapted from [4].

The vascular endothelium is a highly specialised metabolically active organ with several physiological, immune, and synthesizing functions (Table 1). In the present review, we will concentrate on changes that occur in acute COVID-19 and long COVID, in several of these functions (Figure 1).

### 2.1. Barrier and Transport

The endothelium controls the flow of fluid and solutes between the blood and the interstitial space, in addition to serving as a semipermeable barrier separating blood from the tissues or the air (in the lungs). Transport across the endothelium occurs either directly through the endothelial cell (transcellular) or the intercellular junctions (IEJs) between neighbouring cells (paracellular) [8]. In order to support endothelial cell (EC) adhesion to the underlying matrix, IEJs are made up of tight junctions (TJs), adherens junctions (AJs), and gap junctions (GJs), which interact with integrin receptors [9]. TJs represent a selective barrier to the entry of molecules from the circulation, made up of occludin, claudins, and junctional adhesion molecules (JAMs); AJs, made up of vascular endothelial cadherin (VE-cadherin), mediate cell–cell contact, and play a central role in barrier function. GJs, produced from connexions, control the direct transfer of signalling chemicals, ions, and transmembrane potential between cells [9,10].

#### 2.1.1. Vascular Endothelial Cadherin (VE-Cadherin)

Endothelial cells and intercellular connections are controlled by vascular endothelial (VE)-cadherin. It has been proposed that the high circulating levels of VE-cadherin observed in septic patients are a result of endothelial barrier breakdown. These high levels have been associated with worse outcomes [11,12].

Plasma from COVID-19 patients induced a rapid increase in the endothelial permeability of human pulmonary microvascular endothelial cell (HPMEC) monolayers, demonstrated by the loss of junctional VE-cadherin [13]. In another study, and despite the role of VE-cadherin in endothelial function and integrity, no differentiation was found between survivors and non-survivors [14].

In a study published by Patel et al. that included patients with acute COVID-19 and long COVID, there was a significant difference in the levels of VE-cadherin between the acute and post-COVID-19 phase. Patients with long COVID had higher levels of VE-cadherin, compared to both acutely ill patients and healthy controls [15]. This finding suggested that there may be either continued damage to the blood vessels in individuals with long COVID symptoms, or that there is a long-term response of angiogenesis, with a wound-repairing role [15].

#### 2.1.2. Angiopoietins

Angiopoietin-1 (Ang-1) is responsible for maintaining arterial integrity and preventing vascular leakage, whereas angiopoietin-2 (Ang-2) counteracts the protective effects of the Ang-1-Tie2 signalling [16]. These two angiopoietins are antagonistic factors that cause EC activation [17].

Studies have found no difference in Ang-1 levels between severe and critical COVID-19 patients, and survivors and non-survivors [14,18].

On the other hand, Ang-2 has been shown to act as a significant predictor of ICU direct admission in hospitalised COVID-19 patients [19]. Furthermore, it has also been shown to be higher in critically ill COVID-19 patients compared to controls, and a highly effective predictor of in-hospital mortality [20]. In another study, patients who were directly admitted to the ICU rather than the ward had elevated levels of Ang-2, and among the ICU patients, those who eventually died had even higher Ang-2 admission levels [14]. Ang-2 levels have been shown to be elevated in ICU patients [21], in critical COVID-19 patients [18], and in non-survivors [22]. Moreover, concentrations have been shown to be increased with increasing disease severity, and the highest levels were associated with worse survival [23]. Ang-2 levels were significantly higher in the mild–moderate pneumonia and severe/critical patient groups compared to the asymptomatic COVID-19 patients [24]. Other studies have also shown that high Ang-2 levels could predict mortality in patients with COVID-19 [25], while increased levels in COVID-19 patients were correlated with disease severity, hypercoagulation, and mortality [26]. Moreover, proteomic analysis on blood samples from COVID-19 subjects at specific time-points during ARDS pathogenesis revealed a signature of vascular injury, including increased Ang-2 levels, that correlated with low platelet levels and increased mortality [27].

Therefore, the levels of Ang-2, which reflect vascular barrier breakdown, are elevated in COVID-19 patients and can be used as a prognostic factor for poor prognosis. However, treatment with LY3127804, an anti-angiopoietin-2 antibody, in a randomised, double-blind, placebo-controlled clinical trial in COVID-19 pneumonia patients showed no benefit in reducing the need for ventilator usage [28].

In a similar manner to VE-cadherin, the levels of Ang-1 were found to be significantly higher in individuals with long COVID symptoms. In fact, Ang-1 had the highest levels out of 14 biomarkers of vascular injury that were examined. Furthermore, the ROC curve analyses showed that Ang-1 levels had an excellent diagnostic potential, being able to distinguish between long COVID cases and healthy controls, with a high degree of discrimination. It is interesting to note that long COVID patients who received an intervention (inhaled glucocorticoids, anti-coagulants, selective beta 2-adrenergic receptor agonists, diuretics, nasal sprays, physiotherapy, or oxygen) at their follow-up had significantly lower Ang-1 concentrations than those who did not, at approximately 100 days post-acute infection. A possible role of Ang-1 levels in hastening the healing response has been postulated, and this molecule could theoretically be used for the follow-up of long COVID cases. It can, therefore, be suggested that Ang-1 levels may speed up the healing process, and this molecule may be used to monitor long-term COVID-19 cases [15].

There is some conflicting evidence regarding the levels of Ang-2 in the serum of long COVID patients. In a prospective observational study of 215 long COVID patients who were hospitalised at the acute phase of SARS-CoV-2 infection, Ang-2 was found to be significantly elevated at 6 months post-infection in patients complaining of dyspnoea, and in those with severely affected lung diffusing capacity [29]. A non-significant increase was also noted by Fogarty et al. [30]. In another small prospective study, Ang-2 levels were lower in post-COVID-19 patients compared to healthy controls. In the same study, Ang-2 levels were found lower in the post-COVID-19 population that fulfilled the criteria for ME/CFS [31]. Therefore, it is reasonable to assume that decreased Ang-2 levels may help distinguish between long COVID and ME/CFS.

#### 2.1.3. Glycocalyx

The endothelial glycocalyx (EG) is a layer of fibrous matrix, made up from of proteoglycans, glycosaminoglycans, and other glycoproteins, that covers inter-endothelial junctions (IEJs). It is a key regulator of endothelial cell homeostasis, tissue oedema, and inflammatory processes [32]. Microvascular leakage can result from glycocalyx dysfunction, and EG shedding has been observed in flu-infection-induced ARDS [33].

##### Syndecan-1

The main protein found in the glycocalyx is called syndecan-1. When the glycocalyx is damaged, it is released from the endothelium, and increased levels can be found in the blood. Syndecan-1 can regulate leukocyte recruitment, cancer cell growth and invasion, angiogenesis, microbial attachment and entry, host defence systems, and matrix remodelling [34].

In COVID-19 there has been a growing number of studies on the prognostic ability of syndecan-1. Elevated levels of syndecan-1 exist in COVID-19 patients compared to healthy controls [35,36,37]. Moreover, elevated levels have been associated with severe disease [38,39,40,41]. Higher levels have also been related to the risk of death [25,42,43]. Compared to other inflammatory conditions, syndecan-1 levels are higher in severe COVID-19 patients compared to septic shock patients with bacterial pneumonia [44]. All studies agree that syndecan-1 levels are increased in critical illness and may also predict mortality.

In a small observational study of 24 convalescent COVID-19 patients, significantly elevated syndecan-1 levels were detected after a median of 88 days post-symptom onset compared to healthy controls, whereas no difference in syndecan-1 levels was found between the convalescent patients and patients hospitalised for acute disease. This finding implies endothelial damage in convalescent COVID-19 patients who did not require hospitalisation [45]. In the same study, the authors also described a significant correlation between syndecan-1 levels and inflammatory parameters, including lactate dehydrogenase (LDH), ferritin, interleukin (IL)-6, and C-reactive protein (CRP), whereas albumin negatively correlated with syndecan-1 [45]. Another study reported that syndecan-1 levels were not statistically increased compared to controls [46], while a third study reported lower syndecan-1 levels in convalescents compared to the control group [47]. It is, therefore, likely that syndecan-1 levels rise to a lesser degree in long COVID compared to other biomarkers of endothelial injury, such as Ang-2 and VE-cadherin, as described [15].

##### Endocan

The endothelial cell-specific molecule-1, also known as endocan (ESM-1), is produced and secreted by the vascular endothelium in the lungs and kidneys, in response to pro-inflammatory cytokines and pro-angiogenic factors [48]. It is a proteoglycan that affects the ability of leukocytes to roll and migrate through ECs, but does not disrupt their adhesion [49,50]. The molecule is cleaved by cathepsin G, resulting in the production of a 14 kDa novel endocan fragment known as p14, which is elevated in septic patients [50].

Elevated levels of ESM-1 have been detected upon admission in ICU patients compared to ward patients, and ICU non-survivors compared to survivors [51,52]. The prognostic ability, however, of ESM-1 was lost following dexamethasone administration [51].

The higher levels of endocan found in acute COVID-19 patients could: (i) Have added diagnostic benefits in assessing COVID-19 patients; (ii) Be a prognostic marker of adverse outcomes; (iii) Signify worsening of the disease course [53].

Despite higher ESM-1 levels in severe acute COVID-19 disease, ESM-1 does not seem to be relevant in long COVID disease. ESM-1 levels were found to be comparable between long COVID patients compared to healthy controls. Furthermore, no correlation was found between ESM-1 levels and long COVID symptoms, nor with the disruption of the peripheral endothelial function (as assessed by post-occlusive reactive hyperaemia peripheral arterial tonometry—RH-PAT) [31,54].

All studies seem to agree that endothelial injury is one of the most important characteristics of severe acute COVID-19. Vascular endothelial glycocalyx damage can be caused by cytokine production and ROS generation, which are common in COVID-19 [55]. EG shedding causes, in turn, vascular endothelial dysfunction, hyper-permeability, thrombosis, and increased leukocyte adhesion [56], while it can also aggravate the severity of COVID-19 and delay the recovery from vascular dysfunction [57]. It has been shown that heparin can potentially inhibit in vitro glycocalyx shedding induced by COVID-19 patients’ plasma [58].

### 2.2. Host Defence

Communication between cells is essential for immune and inflammatory responses. Communication is facilitated by cytokines, chemokines, interleukins, adhesion molecules, and growth factors.

#### 2.2.1. Cytokines

COVID-19, like other inflammatory conditions, is characterised by a “cytokine storm”. In the presence of viral infection, cytokine-driven vascular leak at the alveolar-endothelial interface of the lung increases acute lung injury. This diffuse alveolar injury results from this excessive immune response [59]. Hence, molecules that attenuate immune dysregulation and hyper-inflammation, and the possible development of a cytokine storm, are being investigated, in an effort to improve the clinical outcomes of COVID-19. In this review we will not discuss the findings on cytokines, as there are numerous reviews on this subject in both acute and long COVID-19 [60,61,62].

#### 2.2.2. Adhesion Molecules

For efficient host defence, circulating polymorphonuclear leukocytes (PMNs) must migrate across the endothelium. The activated neutrophils transmigrate through the endothelium to sites of pathogen invasion or where an injury—physical or toxic—has caused tissue damage.

Selectins (E-selectin and P-selectin) and cell adhesion molecules expressed on the surface of ECs (ICAM-1, PECAM-1, and VCAM-1) are involved in the recruitment of inflammatory cells to the lung. These molecules either develop from de novo synthesis (ICAM-1, PECAM-1, VCAM-1, and E-selectin) or are stored intracellularly and released (P-selectin).

##### The Selectin Family

Prior to their solid adherence and diapedesis at sites of tissue injury and inflammation, activated PMNs adhere to ECs in inflammatory conditions through the selectin family [63]. Circulating leukocytes reversibly bind to the surface of ECs by ligands of the selectin family, which include L-selectin (expressed on leukocytes), E-selectin (expressed on ECs), and P-selectin (ECs and platelets). Being membrane receptors, these ligands act as adhesion receptors to support leukocyte rolling and migration at sites of inflammation. These molecules also exist in a soluble form. The soluble levels of the selectins are present in very low concentrations in healthy humans, so they have been suggested as an alternative-surrogate index for the measurement of endothelial damage or activation [64].

In COVID-19 many studies have investigated soluble (s)E-selectin levels. Elevated levels are higher, as expected, in COVID-19 patients compared to healthy controls [65,66,67]. Furthermore, all related studies have agreed that increased levels are associated with severe disease [14,19,68,69], mortality [14], and worse outcomes [70,71].

Similarly to E-selectin, P-selectin correlates with lung endothelial damage and immunothrombosis. However, it is constitutively present in α-granules of platelets and Weibel–Palade bodies in ECs [72]. Higher sP-selectin levels were detected in COVID-19 patients compared to controls [67], while levels increased with COVID-19 severity [73,74,75], and could prognosticate worse outcome [14,75,76,77]. Elevated levels have been demonstrated in deep vein thrombosis (DVT) development in hospitalised COVID-19 patients [78], and have been suggested to serve as promising predictors for severe COVID-19 infection, thrombosis [79], and thromboembolism [80].

One study, however, has found decreased sP-selectin concentration compared to healthy controls [81]. Their COVID-19 patients showed extensive platelet pathophysiology. The authors explained the presence of low sP-selectin levels in two ways: (i) By suggesting that P-selectin may still be present either on platelets or endothelial cells, acting as an adhesion receptor assisting in platelet activation; (ii) Proposing that sP-selectin is bound to receptors on activated platelets, resulting in decreased levels.

In a previous study from our group, we found no differences in sP-selectin levels in COVID-19 ICU patients compared to non-ICU patients [14]. We suggested that this was due to the fact that we measured the markers upon hospital admission (within 24 h). It is possible that events, such as platelet activation and dysregulation of the coagulation and fibrinolytic systems, may occur as the disease progresses. Similarly, in a very recent study, no significant difference in sP-selectin was observed in the moderate or severe/critical COVID-19 subjects vs. healthy controls [82]. The authors agree that platelet activation in acute COVID-19 is not reflected by sP-selectin levels, due to the sequestration of sP-selectin on adhesion receptors of activated platelets [82].

Since the activation of circulating neutrophils and their transmigration into the alveolar airspace have been associated with the development of ARDS, it has been proposed that inhibitors of neutrophil recruitment may attenuate lung damage [83]. To this end, the effect of a P-selectin inhibitor, crizanlizumab, was assessed in a placebo-controlled, randomised COVID-19 trial [84]. Crizanlizumab was well tolerated; however, no significant differences between crizanlizumab and the placebo group were found for clinical endpoints.

Higher sE-selectin levels in long COVID patients compared to normal controls have been reported in several studies [47,85,86]. Furthermore, E-selectin treatment of whole blood from long COVID patients caused significant platelet activation, plasma protein aggregation, and the formation of microclots [85]. However, no differences in sE-selectin levels were found among patients with normal and reduced lung diffusion capacity 6 months after hospital discharge [29]. This finding in combination with the high levels of sE-selectin during the acute phase, especially in COVID-19-related ARDS [22], suggests that sE-selectin may have a particular role at the initial phase of the pulmonary vasculature damage.

Interestingly, sE-selectin was found to be increased in female long COVID patients compared to male patients, and a possible relationship between E-selectin, coagulation factors, and oestrogen levels has been postulated [86]. The opposite finding has been reported in female patients for sP-selectin, whose levels were lower in long COVID cases compared to normal controls and who exhibited a negative correlation with oestradiol levels [86]. Similarly to Ang-1, sP-selectin was also found to have an excellent correlation with respiratory symptoms and long COVID symptoms [15]. Other investigators, however, have reported that sP-selectin levels did not significantly differ between COVID-19 survivors and healthy controls [87].

##### Soluble Intercellular Adhesion Molecule 1 (sICAM-1)

The intercellular adhesion molecule 1 (ICAM-1) regulates the firm adhesion of neutrophils to the endothelium and assists in their transendothelial migration via the platelet endothelial cell adhesion molecule 1 (PECAM-1) [88].

The soluble form of ICAM-1, sICAM-1, has been extensively studied in relation to COVID-19. Higher sICAM-1 levels have been detected in severe COVID-19 compared to controls [36,41], whereas increased levels have been associated with disease severity, with markedly elevated levels in severe cases [14,89,90]. In cirrhotic patients, elevated plasma levels serve as an independent predictor of severe COVID-19 [91]. sICAM-1 levels have also been associated with increased mortality in COVID-19 [14,22,92]; thus, sICAM-1 might be used both as a predictive factor for ICU admission and mortality and in hospitalised COVID-19 patients.

There is some controversy regarding sICAM-1 levels in long COVID syndrome, with some authors reporting no difference between long COVID cases and normal controls [47], and others reporting increased levels in long COVID cohorts [15,29,93], particularly in patients with respiratory symptoms, such as cough and dyspnoea [29]. Higher sICAM-1 levels have been detected in post-COVID-19 patients, up to one year after acute infection, compared to controls [93], while a predisposition for higher sICAM-1 levels in females compared to males, similarly to sE-selectin, has been described [86].

##### Soluble Platelet Endothelial Cell Adhesion Molecule 1 (sPECAM-1)

Platelet endothelial cell adhesion molecule 1 (PECAM-1/CD31) is a cell adhesion molecule mainly expressed on the surfaces of leukocytes, platelets, and endothelial cells. PECAM-1 constitutes an important component of angiogenesis and inflammation, and takes part in the transendothelial migration of neutrophils [94].

In COVID-19, sPECAM-1 levels have been correlated with disease severity [89,95]. These two studies speculate that endothelial damage is part of the disease’s progression. Admission levels were found to be similar in ICU and ward patients, and in ICU survivors and non-survivors [51]. Therefore, it seems plausible that endotheliopathy progresses with the disease.

Higher levels of sPECAM-1 have also been described in post-COVID-19 cohorts, compared to normal controls, implying a possible role in ongoing endothelial damage after acute infection. However, sPECAM-1 levels did not correlate with long COVID symptoms [15,85].

##### Soluble Vascular Cell Adhesion Molecule 1 (sVCAM-1)

Vascular cell adhesion molecule 1 (VCAM-1/CD106) is a member of the cell adhesion molecules, and its expression is induced by secreted cytokines. VCAM-1 is expressed on the surface of endothelial cells and endothelial cell junctions [96]. Its principal function as a member of the cell adhesion molecules is to mediate leukocyte adherence and recruitment to the endothelium during inflammation [97].

Elevated levels of its soluble form have been observed in patients with severe COVID-19 disease compared to patients with mild disease and controls [36,65,66,89,98,99,100,101]. In critically ill COVID-19 patients, no differences have been found between survivors and non-survivors [22]. In moderate–severe respiratory failure, sVCAM-1 levels were higher in non-survivors [102]. Similar admission levels have been demonstrated in ICU and ward patients; however, higher admission levels in ICU patients could predict mortality [51]. Similarly, elevated levels upon presentation were associated with 30-day mortality [92].

sVCAM-1 was found to be increased in the sera of COVID-19 survivors even after 2 months from the acute infection, while sVCAM-1 levels were found to be higher in men compared to women [86]. Other studies, however, with larger cohorts and longer evaluation intervals since acute infection have reported similar sVCAM-1 values in long COVID patients and controls [47,87]. It can, therefore, be deducted that sVCAM-1 levels return to normal with elapsing time; although, this may take several months.

### 2.3. Angiogenesis

The interaction of growth factors is crucial for vascular development. Vascular endothelial growth factors (VEGFs), angiopoietins, and ephrins have been identified as key participants in several aspects of vascular development [103].

#### 2.3.1. Vascular Endothelial Growth Factor (VEGF)

Vascular endothelial growth factor (VEGF) is a glycoprotein initially identified as a permeability factor with a distinct preference for ECs [104]. It has since been discovered to possess mitogenic and angiogenic activities [105]. VEGF-A regulates angiogenesis and vascular permeability by activating the VEGF receptors (VEGF-R) 1 and 2, whereas VEGF-C and VEGF-D mainly regulate lymphangiogenesis via their receptor VEGF-R3 [105].

VEGF has been found upregulated at the moderate stage of COVID-19 [106], and in COVID-19 patients compared to healthy controls; however, it was unable to predict the appearance of pulmonary embolism during hospitalisation [107]. In another study, high levels could predict mortality in patients with COVID-19 [25]. The VEGF-A molecule has been the focus of the majority of studies investigating the function of VEGF in the lung. To this end, VEGF-A was found significantly elevated in hospitalised COVID-19 patients when compared to mild/moderate cases [108], and elevated in hospitalised patients with non-critical COVID-19 infection [20]. Moreover, VEGF-D levels were significantly higher in the critical group than in the severe group [109]. In other studies, VEGF levels did not correlate with prognosis severity [110] and despite the role of VEGF in endothelial function and integrity, being a potent inducer of vascular permeability, no difference was found between survivors and non-survivors [14]. In COVID-19 individuals with varying degrees of disease severity, elevation of VEGF-R2 expression also occurs, and this might represent compensatory angiogenesis [65].

VEGF is also widely distributed in the brain, and is thought to represent a possible therapeutic target for reducing inflammation in SARS-CoV-2 infection accompanied by neurological symptoms [109]. The preliminary findings of a clinical trial of anti-VEGF for treating patients with severe COVID-19 showed reduced mortality [111].

Compared to healthy controls, recovered COVID-19 patients had higher levels of VEGF-A and VEGF-D, which are associated with endothelial activation. The levels of VEGF-D seemed to increase slowly during the convalescent phase and remained high even at 4 months post-infection [112]. In the same study, VEGF levels did not differ in patients with persistent symptoms, versus those who were symptom-free. Others, however, have reported higher VEGF levels in long COVID patients with shortness of breath [113]. In a smaller study, the soluble VEGF receptor (sVEGF-R) was found to be significantly reduced among long COVID patients. Despite the reduced receptor concentrations, no significant differences in the free VEGF serum levels could be detected [114]. Notably, VEGF has been associated with ME/CFS in the past [115], while down-regulation of sVEGF-R has been previously described in hypoxic conditions and in cases of disturbed microcirculation [116].

#### 2.3.2. Ephrin-A1 and Ephrin Type-A Receptor 2

Ephrin-A1 and ephrin type-A receptor 2 (EphA2) are members of the Eph/ephrin receptor–ligand family. Eph/ephrin contributes to angiogenesis, vascular permeability, and remodelling by regulating endothelial cells and their supporting cells [117]. Ephrin-A1 and EphA2 receptor expression was elevated by inflammatory regulators in in vitro injured lungs, suggesting a role in the control of endothelial permeability [118]. Additionally, it was demonstrated that the EphA2 can cause the elevation of cell adhesion biomarkers via thrombin. All of the aforementioned procedures are crucial to the inflammatory response [119]. A study suggested that ephrin-A1-mediated inflammatory signalling may contribute to COVID-19 disease progression [120]. ICU COVID-19 patients who will eventually die had similar ephrin-A1 and elevated EphA2 levels compared to ICU survivors [51].

### 2.4. Receptors

#### 2.4.1. Triggering Receptor Expressed on Myeloid Cells 1 (TREM-1)

The triggering receptor expressed on myeloid cells 1 (TREM-1) is a member of the immunoglobulin superfamily, primarily expressed on neutrophils, macrophages, and mature monocytes. It acts as an inflammation amplifier and triggers the secretion of pro-inflammatory mediators [121,122]. Apart from immune cells, endothelial cells, vascular smooth muscle cells (VSMCs), and platelets also express TREM-1.

Increased levels of sTREM-1 have been found in patients with COVID-19 compared to healthy controls [123], and in patients with severe disease compared to moderate disease and control groups [124]. sTREM-1 has been proposed as a strong predictive biomarker of COVID-19 severity, and has been related to worse outcomes and death [125]. Levels of sTREM-1 in COVID-19 patients have also been proposed in evaluating the patients’ therapeutic management in the emergency department (ED) [126]. Finally, elevated levels have been found upon admission in ICU patients compared to ward patients, and in ICU non-survivors compared to survivors [51,127].

#### 2.4.2. Soluble CD40 Ligand

CD40 is a transmembrane signalling protein expressed on the surface of B cells, monocytes, dendritic cells, and endothelial cells. CD40 ligation triggers cellular responses that are crucial for immunity and inflammation [128]. The CD40 ligand (CD40L) is a membrane glycoprotein, member of the tumour necrosis factor (TNF) family, mostly secreted by activated T-cells. CD40L is also expressed on human vascular endothelial cells, smooth muscle cells, and macrophages [129]. CD40L plays a central role in pathogen infections, by inducing neutrophil infiltration, increasing the expression of endothelial adhesion molecules, and establishing an inflammatory and coagulant environment [130]. Soluble CD40L (sCD40L) is stored in platelet granules and, hence, its presence in the circulation is a biomarker of platelet activation [131].

In the setting of COVID-19, no difference has been observed in sCD40L levels in ICU, non-ICU patients, non-hospitalised, and asymptomatic controls [73]. ICU non-survivors also exhibited similar admission levels as survivors [51]. However, another study demonstrated higher levels in the COVID-19 cohort compared to controls [36].

There is only one study assessing the levels of sCD40 in a small cohort of long COVID patients and the authors did not find a significant difference in the levels of this biomarker between patients and controls [114].

#### 2.4.3. Soluble Urokinase-Type Plasminogen Activator Receptor (suPAR)

The soluble urokinase-type plasminogen activator receptor (suPAR) is the soluble form of the urokinase plasminogen activator (uPA). It has a key role in the innate host defence in the pulmonary tissue. suPAR levels have been associated with a general immune cell activation rather than with a specific etiological factor [132].

The first study on suPAR in COVID-19 showed that increased plasma levels can act as an early predictor of severe respiratory failure [133]. Subsequently, it was demonstrated that active suPAR may assist in the early triage of SARS-CoV-2-infected persons to prevent virus transmission [134]. Since then, the role of suPAR has been investigated in a number of studies. They have all agreed that suPAR is a very useful prognostic biomarker in COVID-19. More specifically, suPAR has been shown to be elevated in severe versus moderate disease and, furthermore, it has been attributed a prognostic value in identifying patients with a poor prognosis, such as extended stay, or need for mechanical ventilation [133,134,135,136,137,138,139]. Fewer studies have investigated its role in mortality, yet have agreed that higher ICU admission suPAR levels exist in non-survivors compared to survivors [51,140,141].

#### 2.4.4. Presepsin

Cluster of differentiation 14 (CD14) is a glycoprotein expressed on monocytes and macrophages, serving as a pattern recognition receptor that binds directly to lipopolysaccharide (LPS) [142]. It is involved in the innate immune system by activating a pro-inflammatory signalling cascade upon contact with microorganisms [143]. Under inflammatory conditions, soluble CD14 (sCD14) fragments are released by protease activity, one of which has been identified as presepsin (sCD14-ST) [144]. Presepsin is considered a regulator of the immune response and an emerging biomarker of infection.

Most studies have focused on presepsin’s prognostic role as a biomarker of the severity of the disease. It has been found that it may be useful as a prognostic biomarker for severe COVID-19, and those with prolonged hospitalisation [145,146,147,148,149,150]. Very few studies have analysed its role in assessing mortality risk [51,151]. The first study demonstrated that ICU-admission presepsin levels were a valuable prognostic biomarker in assessing ICU mortality risk in COVID-19 patients, even following dexamethasone administration [51], whereas the second study showed that elevated presepsin levels indicated poor outcomes in hospitalised patients with COVID-19 pneumonia and were associated with in-hospital mortality [151].

### 2.5. Coagulation and Fibrinolysis

Coagulation and fibrinolysis are critical host responses to infection and injury involved in COVID-19 pathogenesis. COVID-19 patients have a profound risk for thrombotic and thromboembolic events, due to excessive inflammation, endothelial cell activation and injury, and platelet activation and hypercoagulability. Patients admitted to the ICU have an exceedingly high rate of thromboembolic complications, including DVT and pulmonary embolism, even in the presence of pharmacological thromboprophylaxis. A major role of endothelial cells is to maintain vascular function for blood fluidity and prevent thromboembolic events. Under certain conditions, clot formation (coagulation) should occur for haemostasis. Endothelial cells regulate the immune and haemostatic response by shifting from their normal anti-thrombotic and anti-inflammatory phenotype to an activated state of endothelial dysfunction [4]. To this end, they produce a variety of proteins, including pro-thrombotic substances (von Willebrand factor (vWf) and P-selectin), molecules that limit coagulation (thrombomodulin, nitric oxide, prostacyclin, and tissue factor pathway inhibitor (TFPI)), and fibrinolytic factors (plasminogen activators) [4].

#### 2.5.1. Coagulation

Severe coagulation abnormalities, immune cell infiltration, and platelet activation are found in the autopsy lungs of COVID-19 patients. Pulmonary ECs of COVID-19 patients show increased expression of pro-coagulant vWf and decreased expression of the anticoagulants thrombomodulin (TM) and endothelial protein C receptor (EPCR) [152]. Moreover, transcripts encoding TM and EPCR are reduced in bronchoalveolar lavage fluid (BALF) from COVID-19 patients compared to controls. The authors suggested that SARS-CoV-2 infection correlates with the reduced expression of TM and EPCR on the vascular endothelium, and that increased coagulation and decreased fibrinolysis promote coagulopathy in the lungs of COVID-19 patients [153].

##### Complement System

In COVID-19 patients, upregulation of a pro-coagulant state along with downregulation of the anticoagulant state predisposes them to fibrin clot formation and platelet activation/aggregation. The complement and coagulation systems are functionally dependent. Complement activation is thought to be among the main drivers of COVID-19-associated coagulopathy [154,155]. Indeed, in severe COVID-19, microvascular injury syndrome was shown to be mediated by the activation of complement pathways, providing the basis of the pathophysiologic importance of complement in COVID-19, and proposing targets for specific intervention [156]. Moreover, COVID-19 patients have been shown to demonstrate activation of the complement system, and this has been associated with disease severity, including increased mortality and thromboembolic complications [157]. The authors, hence, suggested that components of the complement system may act as potential prognostic markers for disease severity and as therapeutic targets in COVID-19 [157]. Thus, understanding the potential clinical significance of the complement system in SARS-CoV-2 infection may provide important insight on the pathogenesis of other infections as well. In this review we will not discuss the findings on the complement system and the ongoing clinical trials, as numerous reviews exist on this subject in both acute and long COVID-19 [62,154,155,158].

##### Von Willebrand Factor (vWf)

As mentioned earlier, the expression of adhesion molecules on the EC surface, and the expression of activators of the humoral clotting system, including tissue factor (TF) and von Willebrand factor (vWf), play a central role in the activation of the endothelium [159]. Although the main physiological role of vWf is to activate platelets, it is also considered a sensitive marker of EC injury or activation.

The first studies examining the role of vWf in COVID-19 showed markedly increased plasma vWf levels in patients with increased oxygen requirements [73,160,161,162]. Furthermore, our group found elevated levels in the patients admitted directly to the ICU compared to those admitted to the ward; we also found significantly higher levels in critically ill patients who will eventually die [14].

Since then, many studies have assessed the role of vWf in COVID-19. Higher levels have been found in COVID-19 patients compared to controls [41,65,101], while extremely elevated vWf levels have been seen in all patients, with the highest values in ICU subjects [163]. Increased levels have been detected in individuals with more severe COVID-19 pulmonary disease [74]. Moreover, patients receiving haemodialysis with severe COVID-19 had significantly higher vWf plasma levels [42]. All studies agree that thrombin formation via the intrinsic coagulation pathway and vWf reflect disease severity and poor prognosis in COVID-19 patients [164,165,166,167].

Significantly higher levels of the vWf in long COVID patients compared to controls have been observed in several studies [30,85,93], corroborating a detectable state of hypercoagulability and endothelial dysfunction up to 1 year after acute infection [93]. Nevertheless, it is unclear whether the raised vWf levels specifically represent a more active vWf, since this would require a functional assessment [93]. Other studies have reported that vWf serum levels were not statistically different from controls; however, 6 months after discharge, COVID-19 survivors with abnormal chest computed tomographies and those with severe acute illness had higher vWf levels [168]. This finding supports that persistent symptoms during the recovery period and remaining alveolar injury are related to the presence of high vWf levels [169], and that vWf is also a biomarker of the intensity of the inflammatory response [170].

##### ADAMTS-13

After the findings of greatly elevated vWf levels in COVID-19 patients, studies focused on ADAMTS-13, as well as the balance between vWf and ADAMTS-13. ADAMTS-13, a “disintegrin-like metalloprotease with a thrombospondin type 1 motif, member 13”, cleaves vWf in circulating blood and, thereby, limits platelet thrombosis. Lowering ADAMTS-13 activity leads to accumulation of vWf multimers, which in turn cause clots.

Patients receiving haemodialysis with severe COVID-19 had lower ADAMTS-13 [42]. Deficiency of plasma ADAMTS-13 activity was observed in critical and severe COVID-19 patients compared to healthy controls [37]. Lower ADAMTS-13 activity has been suggested as a marker of poor prognosis [171,172,173,174].

The vWf:ADAMTS-13 ratio has also been used for monitoring the severity of the disease. A decrease in ADAMTS-13 activity results in an increase in vWf concentration and activity, so the ratio of vWf:ADAMTS-13 changes significantly. This imbalance is associated with COVID-19 severity [172,175,176].

ADAMTS-13 levels were reduced, with marked inter-individual variation, and the vWf:ADAMTS-13 ratio was increased in convalescent COVID-19 cases, while levels of platelet factor 4 (PF4), a putative protector of vWf, were also elevated in the same population [30]. ADAMTS-13 levels were significantly lower in patients who had required hospitalisation compared to those managed entirely as outpatients. The absolute reduction in ADAMTS-13 levels in convalescent COVID-19 patients was significantly less marked compared to patients with acute COVID-19. The vWf:ADAMTS-13 ratio was also significantly higher in patients who had required hospitalisation, had ≥2 comorbidities, and those with reduced exercise tolerance. Interestingly, Prasannan et al. also reported similar vWf:ADAMTS-13 ratios in a convalescent COVID-19 cohort in which vWf:ADAMTS-13 ratios were associated with impaired exercise tolerance [177].

##### Tissue Factor (TF)

The coagulation system is a major participant in COVID-19. Tissue factor is the major initiator of the extrinsic coagulation pathway. TF binds and activates factor VII. Activated factor VII (FVIIa) promotes coagulation by activating factor X. Activated factor X (FXa) facilitates the conversion of prothrombin to thrombin [178]. Hence, increased TF expression results in activation of the coagulation cascade, while its expression on monocytes also enhances immunothrombosis [179].

Upregulation of TF expression has been associated with thrombus formation in COVID-19 lungs [180], while post-mortem specimens have demonstrated upregulated TF expression in the lung of fatal COVID-19 cases with loss of thrombomodulin, implying a shift towards a prothrombotic state [181]. Multiple reviews have discussed the potential role of TF expression in response to SARS-CoV-2 infection and have concluded that TF may represent a useful prognostic marker and therapeutic target to reduce thrombosis and inflammation [182,183,184,185,186].

##### Soluble Endothelial Protein C Receptor (sEPCR)

As opposed to tissue factor, the protein C (PC) system provides important control of coagulation. The activated protein C (APC) proteolytically inactivates the cofactors Va and VIIIa. APC is generated by thrombomodulin (TM)-bound thrombin, and the endothelial protein C receptor (EPCR) facilitates this activation [187]. The presence, under normal conditions, of a soluble form of EPCR (sEPCR) that is elevated in inflammatory conditions corroborates the concept of EPCR shedding. Hence, it becomes evident that the membrane-bound EPCR controls PC’s anti-thrombotic and anti-inflammatory pathways, while its soluble form inhibits APC activities, promoting its pro-coagulant effects. sEPCR levels are considered a prognostic factor of DVT [188,189,190,191,192]. Since patients with COVID-19 exhibit a pro-thrombotic or thrombophilic state, our group measured sEPCR levels in COVID-19 patients. Our results showed that sEPCR levels in COVID-19 patients upon hospital admission are considerably elevated compared to outpatients. We proposed that the elevated levels might impair APC’s activities and contribute to the pro-coagulant phenotype [193].

##### Soluble Thrombomodulin

Thrombomodulin (TM) is an anticoagulant proteoglycan located on the EC surface. Activation of PC to APC is catalysed by the thrombin-TM complex; hence, thrombin possesses anticoagulant properties in addition to pro-coagulant properties [194]. The anticoagulant APC strengthens the vascular barrier, while the pro-coagulant thrombin breaks inter-endothelial junctions [4]. The soluble (s)TM fragments released in the blood retain part of their co-factor activity [195]. sTM is less effective in inhibiting fibrinogen and platelet activation and inducing thrombin inhibition compared to endothelial TM [196].

Our group, in an effort to further approach a potential mechanism in the PC pathway in COVID-19, also measured sTM levels along with sEPCR. We observed no difference in sTM levels with respect to the severity of COVID-19, as expressed by ICU/ward hospitalisation, need for intubation, prolonged hospital stay, or mortality [193]. Since then, other studies have also measured sTM levels in COVID-19. Similar levels have been found in severe COVID-19 compared to patients with septic shock due to bacterial pneumonia [44,197]. However, more recent studies have shown that patients with severe and critical COVID-19 have elevated levels compared to healthy controls [37,101], while elevated levels have been demonstrated in COVID-19 ICU patients [21]. Finally, one study suggested that sTM may have predictive ability for mortality in COVID-19 [198].

As with other endothelial biomarkers, sTM levels were significantly elevated in long COVID outpatients, versus acutely ill COVID-19 inpatients and healthy controls [15,199]. A significant correlation between sTM levels and plasma vWf levels was also observed [199]. Another study by the same group also revealed increased intermediate monocytes and activated CD4+ and CD8+ T cell subsets in convalescent COVID-19, which correlated with thrombin generation and endotheliopathy markers, respectively [30].

#### 2.5.2. Platelet–EC Interaction

In addition to endothelial injury and increased thrombin generation, COVID-19 infection is characterised by excessive platelet activation, leading to a hypercoagulable state. The platelet haemostatic plug formed by platelets prevents blood loss, while platelets also serve as a platform for coagulation factors. Platelet adhesion is prevented by an intact endothelium, while it is promoted by activated ECs [200]. Furthermore, platelets also interact with immune cells, promoting haemostasis and inflammation [201,202,203]. The mechanisms by which activated platelets intensify endotheliopathy in COVID-19 are demonstrated in the work by Barrett and co-workers [204]. The mechanisms and events involved in platelet activation and platelet interactions with leukocytes during COVID-19 leading to immunoregulation, inflammation, and hypercoagulability are provided in detail in the reviews by Rossouw et al. and Hottz et al. [205,206].

#### 2.5.3. Fibrinolysis

The pulmonary endothelium actively participates in the fibrinolytic process by secreting plasminogen and its activator inhibitors (tissue-type plasminogen activator (tPA) and urokinase plasminogen activator (uPA)), among others [207].

Alteration in the levels of different factors, including but not limited to, plasminogen activator inhibitor 1 (PAI-1), vWf, sTM, and TFPI seems to cause the hypercoagulation state induced by EC dysfunction [208]. A meta-analysis showed that higher plasma levels of vWf antigen, tPA, PAI-1, and sTM were associated with composite poor outcome in COVID-19 patients [209].

##### Plasminogen

Plasminogen, the zymogen of plasmin, proteolytically degrades excess fibrin, resulting in elevated fibrin degradation products.

Patients with hypertension, diabetes, coronary heart disease, cerebrovascular illness, chronic obstructive pulmonary disease, and kidney dysfunction have worse clinical outcomes when infected with SARS-CoV-2. A review summarised the evidence for the existence of elevated plasmin(ogen) in comorbid COVID-19 patients at a higher risk of worse outcomes [210]. The authors proposed that plasmin could cleave a newly inserted furin site in the spike (S) protein of the SARS-CoV-2 virus, increasing its infectivity and virulence. Hyperfibrinolysis associated with plasmin leads to elevated D-dimer concentrations in severe patients; hence, the authors suggest that plasmin(ogen) may prove to be a promising therapeutic target for combating COVID-19 [210].

In this respect, several studies assessed plasminogen levels in COVID-19. These investigations found either that COVID-19 patients had similar levels with non-COVID-19 sick controls, while ICU-admitted patients had lower values compared to ward-admitted patients [211], or that ICU non-survivors had lower values compared to survivors, suggesting that plasminogen may have predictive ability for mortality in COVID-19 [198]. However, one study demonstrated that non-survivors had higher levels of plasminogen compared to survivors, who had not received dexamethasone treatment. Administration of dexamethasone, however, resulted in decreased levels of plasminogen and loss of its prognostic value [51].

##### Plasminogen Activator Inhibitor 1 (PAI-1)

Plasminogen activator inhibitor 1 (PAI-1), also known as SERPIN 1, is the major inhibitor of fibrinolysis; its upregulation results in a shift from pro- to anti-fibrinolytic phenotypes [212].

One of the first studies of PAI-1 in COVID-19, showed no difference in ICU patients, non-ICU patients, and non-hospitalised, asymptomatic controls [73]. Subsequently, PAI-1 levels were found to be elevated in patients with critical COVID-19 infection, and were strongly predictive of in-hospital mortality [20]. Others have since also observed markedly elevated levels in patients hospitalised with COVID-19 and have associated them with poor outcomes [165,198,213,214,215,216]. One study has demonstrated decreased levels compared to normal controls [66], whereas in a study by our group, levels of PAI-1 were not indicative of mortality in critically ill patients [51]. Al-Tamini and co-workers proposed that the induction of PAI-1 suggests the activation of platelets and the coagulation system at the moderate stage before COVID-19 patients require intensive care [217].

Elevated D-dimers and fibrinogen levels are a hallmark of COVID-19 patients. The dysregulation of coagulation and fibrinolysis is most likely the cause of the fibrin deposits discovered in the patients’ lungs. The accumulated data until now indicate that fibrinolytic homeostasis in COVID-19 is complex, with a subset of patients expressing a balance of factors that may favour fibrinolysis. The findings of elevated PAI-1 levels in patients with severe COVID-19 suggest dysregulation of the fibrinolytic system and a phenomenon of “fibrinolytic shutdown” or “temporary exhaustion” in COVID-19 patients [213]. In another study, fibrinogen, tPA, and PAI-1, but not plasminogen levels, were elevated in patients with severe COVID-19. The authors suggested that hypercoagulability in COVID-19 may be due to decreased fibrinolysis resulting from the inhibition of plasmin through high levels of PAI-1 [218]. In agreement with this suggestion, another study demonstrated that increased PAI-1 levels from the lung epithelium and ECs promote a hypofibrinolytic condition attenuating plasmin generation, whereas damaged endothelial cells and leukocytes expose TF and promote fibrin deposition [219]. Thus, it has been proposed that nebuliser plasminogen activators, including pre-existing drugs, such as Tenecteplase, a modified recombinant tPA molecule, may be used as a treatment for COVID-19 patients, by facilitating fibrin degradation [220].

Other therapeutic options enhancing fibrinolysis have also been explored. Antithrombin, also termed antithrombin III (ATIII), is a small glycoprotein that inactivates several enzymes implicated in the coagulation cascade. Its activity increases when bound to the anticoagulant drug heparin. Argatroban, a direct thrombin inhibitor, successfully treated severe COVID-19 patients with acute ATIII deficiency, with confirmed thrombosis upon hospitalisation and resistance to the use of a prophylactic dose of low molecular weight heparin (LMWH). It seems that ATIII deficiency may contribute both to the development of thrombosis and failure to achieve therapeutic anticoagulation with heparin [221]. Moreover, in another study following argatroban initiation, D-dimer levels decreased but remained high, while fibrinogen levels return to normal. Argatroban, unlike heparin, could enhance fibrinolysis via the inhibition of the thrombin activatable fibrinolysis inhibitor (TAFI), and a reduced activation of factor XIII [222].

The plasmin–antiplasmin system also has a central role in coagulation and fibrinolysis. Plasmin and α(2)-antiplasmin are primarily responsible for the breakdown of fibrin polymers into soluble fragments [223]. Acute COVID-19 patients exhibit the deposition of microclots in the lungs. A study showed that plasma samples from long COVID patients still contain large amyloid deposits, which were resistant to fibrinolysis, even with trypsinisation [224]. Hence, the authors suggested that coagulation abnormalities seen in both acute COVID-19 and long COVID might benefit from anticlotting therapy to support fibrinolysis [224].

To summarise, coagulation (as measured by plasma levels of protein C) and fibrinolysis (as measured by plasma levels of PAI-1) have been shown to be markedly abnormal in COVID-19 and have been independently associated with adverse clinical outcomes.

### 2.6. Maintenance of Vascular Tone

The vascular endothelium has an important metabolic function, synthesising, among others, several vasoconstrictors and vasodilators, including endothelin-1, angiotensin II, nitric oxide, and prostacyclin, which regulate vasomotor tone, the recruitment and activity of inflammatory cells, proliferation, and thrombosis [225]. In ARDS, the balance shifts from pulmonary vasodilators towards vasoconstrictors, resulting in increased pulmonary vascular resistance and pulmonary hypertension [226].

#### 2.6.1. Endothelin-1

Endothelin-1 (ET-1), a potent vasoconstrictor peptide produced by endothelial cells, has been implicated in the development of lung injury.

Very few studies have measured endothelin-1 in COVID-19. Increased levels have been found in the plasma of patients hospitalised with COVID-19 [227], while auto-antibodies against the endothelin-1 type A receptor (ETAR) have been found to be significantly increased in COVID-19 patients with an unfavourable disease course [228].

ET-1 concentration was significantly elevated in both long COVID syndrome cases and the subgroup of cases compatible with ME/CFS compared to healthy controls [31]. However, its levels did not correlate with peripheral endothelial function, as assessed by RH-PAT [31,229].

#### 2.6.2. Renin–Angiotensin–Aldosterone System (RAAS)

The main enzyme of the Renin–Angiotensin–Aldosterone System (RAAS), the angiotensin-converting enzyme (ACE), hydrolyses angiotensin I into angiotensin II and breaks down bradykinin, whereas ACE2 converts angiotensin II into angiotensin (1–7). Angiotensin II is a potent vasoconstrictor with pro-fibrotic and pro-inflammatory properties, whereas angiotensin I is a powerful vasodilator with anti-apoptotic and anti-proliferative properties [230,231]. Therefore, ACE2 is considered to be a negative regulator of the classical ACE [232].

From the start of the pandemic, it was demonstrated that ACE2 acts as the receptor for the SARS-CoV-2 entry into the host; hence, cells with increased ACE2 expression had a greater probability of being infected. As mentioned above, this axis has a deleterious arm (ACE) and a protective arm (ACE2). Infection with SARS-CoV-2 mediates the downregulation of ACE2 and interrupts the equilibrium between ACE and ACE2, by removing the protective arm, and, hence, increases angiotensin II [233]. Soluble (s)ACE2, the shed isoform of ACE2, preserves its catalytic activity; however, its role in viral entry remains as yet unclear. Despite the beneficial effect of ACE2, conflicting results on sACE2 levels in COVID-19 have been published. Elevated sACE2 levels in one critically ill COVID-19 patient with ARDS impelled the authors to propose sACE2 as an endogenous non-specific protective mechanism against SARS-CoV-2 infection [234]. The therapeutic value of sACE2 could be exerted by reducing the levels of the vasoconstrictor angiotensin II, and protecting against SARS-CoV-2 by blocking its binding sites for the membrane ACE2 receptor [234]. However, in other studies severe patients with high sACE2 levels do not seem to gain a benefit in viral infection [235,236]. This possible detrimental role of increased sACE2 levels could be attributed to the binding of SARS-CoV-2 to many sACE2 molecules; the virus-sACE2 complex might cause the virus to spread to distant organs, thus creating a depletion of sACE2 and membrane ACE2. The resulting increase of angiotensin II is part of the overall RAAS pathology, and might aggravate the disease [237]. Other published papers have shown no change, or even decreased sACE2 in severe COVID-19 patients compared to pre-pandemic controls [238]. Hence, to ensure the safe and efficient use of therapeutic sACE2 it is of outmost importance to monitor both sACE2 and angiotensin II levels in COVID-19 patients. It is also worth noticing that angiotensin II can downregulate endothelial nitric oxide synthase (eNOS) protein expression via an angiotensin II type 1 receptor-linked pathway [239]. A recent comprehensive review explored the relationship between sACE2 and COVID-19, and the authors concluded that it is still unclear how SARS-CoV-2 infection and recovery affect sACE2 levels [240].

ACE2 seems like an ideal target for therapy. A catalytically active engineered receptor has been suggested to limit the potential for viral escape [241]. Indeed, human recombinant sACE2 was able to neutralise COVID-19 infection by acting as a decoy receptor and, thus, preventing viral entry in target cells [242]. These recombinant molecules compete with membrane-bound ACE2 for SARS-CoV-2 binding, leaving ACE2 on endothelial cell surfaces unoccupied and able to convert angiotensin II to angiotensin (1–7), thus moderating the excessive inflammatory response seen in COVID-19 [243].

The deleterious arm of the RAAS axis is ACE. Baseline serum ACE activity of severe and non-severe COVID-19 patients was reduced compared to normal controls, with the lowest levels seen in the severe COVID-19 group [244]. The respective activities, however, increased in the recovery phase, and the authors concluded that serum ACE activity could be potentially used as a marker reflecting COVID-19 severity, since low activity was associated with the severity of COVID-19 at baseline, and the activity increased with the remission of the disease [244]. Likewise, others have shown that serum ACE activity was lower in patients with COVID-19 vs. controls [245,246]. In another study, serum ACE activity upon hospital admission did not reflect disease severity [247], while our group demonstrated that serum ACE activity was similar in COVID-19 ARDS and non-ARDS patients [236].

ACE genotypes have been also linked to an increased risk for severe COVID-19 disease [248]. Most studies agree that a higher link exists between the ACE deletion/deletion (D/D) genotype and severe COVID-19 [246,249,250].

There is little evidence on the role of ACE and ACE2 in long COVID pathophysiology. One study reported similar levels of sACE and sACE2 in long COVID patients and normal controls, while no correlation was apparent between sACE levels and the assessment of endothelial function [31,229].

#### 2.6.3. Endothelial Nitric Oxide Synthase (eNOS)

Nitric oxide (NO) is an effective vasodilator and inhibitor of vasoconstriction and platelet aggregation. Endothelial nitric oxide synthase (eNOS) is an enzyme that is abundantly expressed in the lung and constitutively produces NO. The increased mortality risk associated with diseases with underlying endothelial dysfunction, such as ARDS, suggests that NO derived from eNOS could represent an important defence mechanism. Indeed, NO, the main intracellular antiviral defence, has been shown to inhibit SARS-CoV, amongst others [251].

To our knowledge, only one study has measured eNOS levels in COVID-19 patients. The results showed that patients with COVID-19-induced ARDS had lower soluble eNOS levels, suggesting that the presumed higher eNOS activity and resulting increased NO synthesis, might protect patients from severe lung complications [236].

The therapeutic use of inhaled (i)NO has also been tested in COVID-19. iNO delayed respiratory deterioration in COVID-19-induced moderate to severe ARDS [252], improved oxygenation parameters, but showed no benefit on mortality [253]. In another study, iNO marginally improved oxygenation in COVID-19-related ARDS [254] [26]. On the contrary, it did not improve oxygenation in COVID-19 patients with refractory hypoxemia [255].

#### 2.6.4. Adrenomedullin

Adrenomedullin (ADM) is a circulating peptide hormone with vasodilatory effects. It is essential for maintaining vascular tone and endothelial barrier function. The ADM plasma levels are markedly increased during severe inflammatory disorders, including COVID-19, and are associated with the severity and prognosis of the inflammatory condition [256].

The biologically active adrenomedullin (ADM) is an emerging biomarker of endothelial function and integrity with a proven utility in interstitial pneumonia upon ED admission [257]. Most studies, however, in COVID-19 have focused on the more stable part of the ADM precursor peptide, mid-regional pro-adrenomedullin (MR-proADM). MR-proADM prevents endothelial permeability and has been associated with a poor prognosis in bacterial sepsis [258]. Studies with the bioactive molecule are scarce. ADM RNA expression is increased in patients with severe COVID-19 [259]. Non-survivors showed significantly higher ADM levels than survivors and, furthermore, ADM levels predicted 28-day mortality [260]. In COVID-19 patients, ADM was a good predictor of patient mortality [257]. ADM responses were associated with short-term mortality in critically ill COVID-19 patients [261].

In an experimental animal model, exogenous ADM administration reduced inflammation and related organ damage. These data prompted the authors to suggest that ADM may be a potential therapy in COVID-19 [256]. In agreement with this, the administration of adrecizumab (HAM8101), a monoclonal anti-ADM antibody targeting the inflammation-based vascular leakage, in critically ill COVID-19 patients with ARDS resulted in a favourable outcome [262]. These findings suggest that treatment with specific antibodies modulating ADM-related pathways may improve the outcome of COVID-19.

Table 2 summarises the findings on the endothelial biomarkers involved in the acute phase of COVID-19 and long COVID.

**Figure 2:**
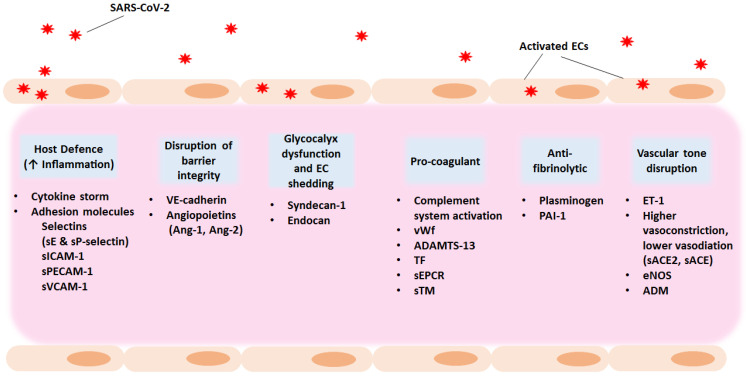
Potential therapeutic strategies for COVID-19-associated endotheliopathy and coagulopathy. Exploring host targets as well as anti-inflammatory and anti-coagulant agents as part of the treatment strategy could benefit hospitalised patients and give relief to individuals suffering from long COVID manifestations. The entry of SARS-CoV-2 into the host results in increased vascular permeability and endothelial cell activation or dysfunction causing a generalised inflammatory state. Main characteristics are the high levels of cytokines, vWf, and endothelial cell adhesion molecules, promoting coagulation and leukocyte recruitment. The increase in PAI-1 levels results in hypofibrinolysis. ACE, angiotensin converting enzyme; ADM, adrenomedullin; Ang-1, angiopoietin-1; Ang-2, angiopoietin-2; EC, endothelial cell; eNOS, endothelial nitric oxide synthase; ET-1, endothelin-1; PAI-1, plasminogen activator inhibitor 1; RAAS, Renin–Angiotensin–Aldosterone System; SARS-CoV-2, severe acute respiratory syndrome coronavirus 2; sEPCR, soluble endothelial protein C receptor; sICAM-1, soluble intercellular adhesion molecule 1; sPECAM-1, soluble platelet-endothelial cell adhesion molecule 1; sTM, soluble thrombomodulin; suPAR, soluble urokinase-type plasminogen activator receptor; sVCAM-1, soluble vascular adhesion molecule 1; TF, tissue factor; VE- cadherin, vascular endothelial cadherin; vWf, von Willebrand factor.

It becomes evident that SARS-CoV-2 infection results in increased vascular permeability and endothelial cell activation/dysfunction causing a generalised inflammatory state. Main characteristics are the high levels of cytokines, vWf, and endothelial cell adhesion molecules, promoting coagulation, leukocyte recruitment, and hypofibrinolysis. Thus, apart from focusing on viral targets as antiviral therapeutic candidates, exploring anti-inflammatory and anti-coagulant agents as part of the treatment strategy could benefit hospitalised patients and give relief to individuals suffering from long COVID manifestations. Figure 2 outlines potential therapeutic strategies for COVID-19-associated endotheliopathy and coagulopathy.

## 3. Conclusions

The endothelium is highly affected and partly damaged in severe COVID-19. Under normal conditions, the resting pulmonary endothelium supports the maintenance of fibrinolysis [65]. However, the presence of pro-inflammatory mediators drives a pro-coagulant state that finally leads to the over-activation of coagulation and thrombosis. Platelet adhesion and thrombus formation are among the most prominent features of COVID-19, as demonstrated by increased fibrinogen, and various markers of endothelial cell and platelet activation, including vWf, sTM, sCD40L, PAI-1, and sP-selectin. Elevated levels of vWf and PAI-1, and decreased ADAMTS-13 activity, demonstrate that COVID-19 is an endotheliopathy that shares features with thrombotic microangiopathy [174]. The release of vWf from Weibel–Palade bodies, which ensures rapid reaction to the damage and recruitment of inflammatory cells, further highlights endothelial activation in critically ill COVID-19 patients. The principal molecules stored and rapidly released from Weibel–Palade bodies by exocytosis are vWf, Ang-2, and P-selectin, all of which are elevated in critically ill COVID-19 patients, implying early dysregulation of haemostasis and inflammation. Thus, targeting exocytosis might contribute to the management of inflammatory or thrombotic conditions seen in COVID-19.

Apart from platelet adhesion and coagulation, endothelial dysfunction, as depicted by the increased levels of various adhesion molecules, the breakdown of the protective glycocalyx, and impaired vascular barrier function constitutes another main characteristic of the COVID-19 pathobiology. In COVID-19 individuals with varying degrees of disease severity the elevation of VEGF-R2 expression also occurs, and this might represent compensatory angiogenesis [65].

Continuous endothelial involvement also seems likely to be an underlying mechanism in the long COVID syndrome. A number of biomarkers of endothelial damage have been studied and found to be affected in long COVID cases, even when a significant amount of time has elapsed since acute illness. Among these biomarkers, Ang-1, Ang-2, sP-selectin, sICAM-1, VEGF, and vWf are the most promising markers related to the presence of long COVID symptoms, which could be evaluated further for their diagnostic value and as therapeutic targets in long COVID disease.

The COVID-19 pandemic highlighted the importance, as well as the challenges, of discovering and designing effective and innovative prophylactic and treatment strategies. Novel therapeutic interventions should not only be restricted to the acute phase of COVID-19 but should also be explored for long COVID treatment. Rapidly emerging data on COVID-19 are providing insights into how endothelial dysfunction may contribute to the pandemic. This may lead to the search for prognostic biomarkers, as well as treatments targeting pathogenic endothelial responses.

## Figures and Tables

**Table 1:** Major endothelial functions.

Barrier and Transport
Host defence
Expression of adhesion molecules
Angiogenesis
Expression of receptors, enzymes, and signal transduction molecules
Coagulation and Fibrinolysis
Maintenance of vascular tone
Production of reactive oxygen species (ROS)

**Table 2:** Endothelial biomarkers involved in the acute phase of COVID-19 and long COVID.

Biomarker	Biological Function	Studies in Acute COVID-19	Studies in Long COVID
VE-cadherin	Controls the structure of intercellular junctions and endothelial cells.	No difference between ICU and ward patients, and ICU survivors vs. non-survivors [14]. Plasma from COVID-19 patients induced a rapid increase in endothelial permeability of human pulmonary microvascular endothelial cell monolayers, demonstrated by the loss of junctional VE-cadherin [13].	Higher levels in long COVID cases compared to acute illness and healthy controls [15].
Ang-1	Preserves vessel integrity and inhibits vascular leakage.	No difference between ICU and ward patients, and survivors and non-survivors [14]. Levels did not differ between severe and critical COVID-19 patients [18].	Increased levels in long COVID compared to controls, with high discriminative ability for long COVID syndrome [15].
Ang-2	Promotes vascular barrier breakdown.	Can predict ICU admission in hospitalised COVID-19 patients [19]. Increased in critically ill COVID-19 patients compared to controls, and predictive of in-hospital mortality [20]. Elevated levels upon admission in ICU patients compared to ward patients, and ICU non-survivors had higher admission levels than survivors [14]. Ang-2 levels have been shown to be elevated in ICU patients [21], in critical COVID-19 patients [18], and in non-survivors [22]. Moreover, concentrations have been shown to be increased with increasing disease severity, and the highest levels were associated with worse survival [23]. Ang-2 levels were significantly higher in the mild–moderate pneumonia and severe/critical patient groups compared to the asymptomatic and non-complicated COVID-19 patients [24]. Other studies have also shown that high Ang-2 levels could predict mortality in patients with COVID-19 [25], while increased levels in COVID-19 patients were correlated with disease severity, hypercoagulation, and mortality [26].	Reduced Ang-2 in post-COVID-19 ME/CFS [31], increased levels in long COVID cases with abnormal lung diffusion capacity [29]. Non-significant increase [30].
Syndecan-1	The core protein in heparan sulphate proteoglycan that is observed in the glycocalyx.	Elevated levels of syndecan-1 in COVID-19 patients compared to healthy controls [35,36,37]. Moreover, elevated levels have been associated with severe disease [38,39,40,41]. Higher levels have also been related to the risk of death [25,42,43]. Compared to other inflammatory conditions, syndecan-1 levels are higher in severe COVID-19 compared to septic shock patients with bacterial pneumonia [44].	Higher [15,45], lower [47], or similar [46] levels compared to healthy controls.
Endocan	Expressed by vascular endothelial cells. It affects leukocytes’ ability to roll and migrate through endothelial cells but does not disrupt their adhesion.	Elevated levels upon admission in ICU patients compared to ward patients, and ICU non-survivors had higher admission levels than survivors [51,52]. The prognostic ability, however, of ESM-1 was lost following dexamethasone administration [51].	Not affected in long COVID [31].
sE-selectin	Early mediator of the adhesion of activated neutrophils to endothelia in inflammatory states, exclusively expressed on activated endothelial cells.	Elevated levels are higher in COVID-19 patients compared to healthy controls [65,66,67]. Increased levels in COVID-19 patients directly transferred to the ICU compared to patients who were admitted in conventional wards [19]. Increased levels in patients with severe compared to mild disease [68]. ICU non-survivors have higher admission levels than survivors [14]. Increased levels upon hospital admission associated with critical disease [69]. sE-selectin is an independent indicator of a worse prognosis in COVID-19 patients requiring hospitalisation [70]. sE-selectin levels are predictive of ICU admission in COVID-19 patients [71].	Higher levels [47,85,86].
sP-selectin	Early mediator of the adhesion of activated neutrophils to endothelia in inflammatory states, constitutively expressed in lung endothelial cells and activated platelets.	Elevated levels in COVID-19 ICU patients compared to non-ICU patients [73]. Similar levels in ICU and ward patients; however, ICU non-survivors had higher admission levels than survivors [14]. Survivors had significantly lower levels compared to non-survivors [77]. Elevated levels in deep vein thrombosis development in hospitalised COVID-19 patients [78]. Promising predictors for severe COVID-19 infection and predictable thrombosis [79]. Increased levels of in individuals with more severe COVID-19 pulmonary disease [74]. Marker of thromboembolism [80]. Elevated levels associated with severity and in-hospital mortality [75]. sP-selectin associated with all-cause mortality [76]. Decreased sP-selectin concentration in COVID-19 patients compared to healthy controls [81]. Higher levels of sP-selectin were detected in COVID-19 patients compared to controls [67]. No significant difference in the moderate or severe/critical COVID-19 subjects vs. healthy controls [82].	Lower levels in long COVID [86]. Significant correlation with long COVID symptoms [15]. No difference between long COVID and controls [87].
sICAM-1	Controls the firm adhesion of neutrophils on endothelium and, consequently, transendothelial neutrophil migration response to sites of infection.	Higher levels in severe COVID-19 patients compared to controls [41]. Higher levels in the COVID-19 cohort compared to controls [36]. Elevated levels in severe cases in ward patients [89]. Elevated levels upon admission in ICU patients compared to ward patients, and ICU non-survivors had higher admission levels than survivors [14]. Higher levels in non-survivors [22]. Higher levels in severe cases [90]. In patients with cirrhosis, elevated plasma levels serve as an independent predictor of severe COVID-19 [91]. Elevated levels upon presentation associated with the composite outcome of ICU admission or 30-day mortality in COVID-19 [92].	No difference between long COVID cases and normal controls [47]. Increased levels in long COVID cohorts [15,29,93].
sPECAM-1	Integral membrane glycoprotein, mainly expressed in endothelial cells, platelets, and leukocytes. Comprises an important component in angiogenesis and inflammation and participates in the transendothelial migration of neutrophils.	Levels have been correlated with disease severity [89,95]. Admission levels similar in ICU and ward patients, and also in ICU survivors and non-survivors [51].	Higher levels in long COVID cases than normal controls [15,85].
sVCAM-1	Member of the cell adhesion molecules. Its primary role is to mediate the adhesion and recruitment of leukocytes to the endothelium during inflammation.	Elevated levels in patients with severe disease compared to patients with mild disease and controls [36,65,66,89,98,99,100,101]. In critically ill patients, no differences between survivors and non-survivors [22]. In COVID-19 patients with moderate–severe respiratory failure, sVCAM-1 levels were higher in non-survivors [102]. Similar admission levels in ICU and ward patients; however, higher admission levels in ICU non-survivors compared to survivors [51]. Elevated levels upon presentation were associated with the composite outcome of ICU admission or 30-day mortality in COVID-19 [92].	Either higher [86], or similar [15,47,87] levels.
VEGF	Glycoprotein originally isolated as a permeability factor with unique specificity for vascular endothelial cells but was subsequently shown to have mitogenic and angiogenic properties.	Upregulated at the moderate stage of COVID-19 [106]. Levels elevated in COVID-19 patients compared to healthy controls; however, unable to predict the appearance of pulmonary embolism during hospitalisation [107]. High levels predicted mortality in patients with COVID-19 [25]. VEGF-A elevated in hospitalised patients with non-critical COVID-19 infection [20], and elevated in hospitalised COVID-19 patients when compared to mild/moderate cases [108]. VEGF-D levels higher in the critical group than in the severe group [109]. In other studies, VEGF levels did not correlate with prognosis severity [110], while no difference was found between survivors and non-survivors [14]. In COVID-19 individuals with varying degrees of disease severity, elevation of VEGF-R2 expression [65].	Either increased [113] or similar [114] VEGF levels in long COVID cases compared to controls. Compared to healthy controls, recovered COVID-19 patients had higher levels of VEGF-A and VEGF-D, but similar in patients with persistent symptoms versus those who were symptom-free [112]. sVEGF-R was found to be significantly reduced among long COVID patients [114].
Ephrin-A1 and EphA2	Part of the Eph/ephrin receptor–ligand family; participate in basic developmental processes and cell activities that depend on their interaction. They are constitutively expressed in pulmonary vascular cells and participate in angiogenesis.	Εphrin-A1-mediated inflammatory signalling may contribute to COVID-19 disease progression [120]. Ephrin-A1 admission levels similar in ICU and ward patients, and also in ICU survivors and non-survivors [51]. ICU COVID-19 patients who will eventually die have elevated EphA2 levels compared to ICU survivors [51].	-
sTREM-1	Member of the immunoglobulin superfamily; expressed on neutrophils, macrophages, and mature monocytes. Acts as an inflammation amplifier, triggering the secretion of pro-inflammatory mediators.	Increased levels of sTREM-1 have been found in patients with COVID-19 compared to healthy controls [123], and in patients with severe disease compared to moderate disease and control groups [124]. Elevated levels upon admission in ICU patients compared to ward patients, and ICU non-survivors had higher admission levels than survivors [51,127]. Strong predictive biomarker of COVID-19 severity and related to a worse outcome and death [125]. Levels of sTREM-1 in COVID-19 patients can evaluate the patients’ therapeutic management in the emergency department [126].	-
sCD40L	Member of the tumour necrosis factor (TNF) family. Its soluble form (sCD40L) is mainly secreted from activated platelets.	No difference in sCD40L levels in ICU patients, non-ICU patients, and non-hospitalised, asymptomatic controls [73]. ICU non-survivors also exhibited the same admission levels as survivors [51]. Higher levels in the COVID-19 cohort compared to controls [36].	No difference between long COVID patients and controls [114].
suPAR	Soluble form of the urokinase plasminogen activator. Plays an important role in the innate host defence in the pulmonary tissue. suPAR levels have been associated with a general activation of the immune system rather than with a particular etiological factor.	Increased plasma suPAR levels in COVID-19 can act as an early predictor of severe respiratory failure [133]. Active suPAR may assist in the early triage of SARS-CoV-2-infected persons to prevent virus transmission [134]. Elevated in severe versus moderate disease and, furthermore, has been attributed a prognostic value in identifying patients with a poor prognosis [133,134,135,136,137,138,139]. Higher ICU admission suPAR levels in non-survivors compared to survivors [51,140,141].	-
Presepsin	Emerging biomarker of infection. Regulates the immune response.	Prognostic biomarker for severe COVID-19, and those with prolonged hospitalisation [145,146,147,148,149,150]. ICU admission presepsin levels were a valuable prognostic biomarker for ICU mortality risk in COVID-19 patients, even following dexamethasone administration [51]. Elevated presepsin levels indicated poor outcomes in hospitalised patients with COVID-19 pneumonia and were associated with in-hospital mortality [151].	-
vWf	Endothelial product that mediates platelet adhesion at sites of vascular damage.	Markedly increased plasma vWf levels in patients with increased oxygen requirements [73,160,161,162]. Increased admission levels in the ICU-admitted patients compared to those admitted to the ward and, furthermore, significantly higher levels in critically ill non-survivors compared to survivors [14]. Elevated levels in COVID-19 patients compared to healthy controls [65,101]. Higher levels in severe COVID-19 patients compared to controls [41]. Extremely elevated vWf levels in all patients with highest values in ICU subjects [163]. Increased levels in individuals with more severe COVID-19 pulmonary disease [74]. Significantly higher plasma vWf levels in severe COVID-19 patients receiving haemodialysis [42]. Increased vWf levels reflect disease severity and a poor prognosis in COVID-19 patients [164,165,166,167].	Increased in long COVID [85,93,199]. Increased levels only in cases with follow-up chest computed tomography abnormalities [168].
ADAMTS-13	A metalloproteinase that specifically cleaves the large multimers of vWf.	Patients receiving haemodialysis with severe COVID-19 had lower ADAMTS-13 [42]. Deficiency of plasma ADAMTS-13 activity in critical and severe COVID-19 patients compared to healthy controls [37]. Lower ADAMTS-13 activity is a marker of poor prognosis [171,172,173,174].	Reduced in long COVID [30].
TF	Major initiator of the extrinsic coagulation pathway.	Upregulation is associated with thrombus formation in COVID-19 lungs [180]. Upregulation of pulmonary TF in COVID-19 patients [181].	-
sEPCR	Conversion of PC to activated PC (APC) is drastically augmented by the presence of EPCR. The soluble form of EPCR (sEPCR) is elevated in conditions marked by enhanced inflammation.	sEPCR levels in COVID-19 patients upon hospital admission are considerably elevated compared to outpatients [193].	-
sTM	Thrombomodulin is an anticoagulant proteoglycan located on the EC surface, which reacts with thrombin producing a marked increase in the thrombin-catalysed activation of protein C.	Similar sTM levels irrespective of COVID-19 severity, as expressed by ICU/ward hospitalisation, mechanical ventilation requirement, prolonged stay, or mortality [193]. Similar levels in severe COVID-19 compared to patients with septic shock due to bacterial pneumonia [44,197]. Patients with severe and critical COVID-19 had elevated levels compared to healthy controls [37,101]. Elevated in ICU patients [21]. sTM may have predictive ability for mortality in COVID-19 [198].	Elevated in long COVID compared to acutely ill and healthy controls [15,199].
Plasminogen	Proteolytically breaks down excess fibrin to elevate fibrin degradation products in both bronchoalveolar lavage fluid and plasma.	Similar levels in COVID-19 patients with non-COVID-19 sick controls, while patients admitted to the ICU exhibited lower values compared to patients admitted to the ward [211]. ICU non-survivors had lower values compared to survivors, suggesting that plasminogen may have predictive ability for mortality in COVID-19 [198]. Higher levels in non-survivors compared to survivors; however, dexamethasone treatment resulted in decreased levels of plasminogen and loss of its prognostic value [51].	-
PAI-1	Major inhibitor of fibrinolysis, whose upregulation leads to a shift from pro- to anti-fibrinolytic phenotypes.	Similar levels in ICU patients, non-ICU patients, and non-hospitalised, asymptomatic controls [73]. Elevated in critically ill COVID-19 patients, and strongly predictive of in-hospital mortality [20]. May have predictive ability for mortality in COVID-19 [198]. Markedly elevated levels in patients hospitalised with COVID-19 and associated with worse outcomes [165,213,214,215,216]. Levels not indicative of mortality [51]. PAI-1 induction suggests the activation of platelets and the coagulation system at the moderate stage of the disease prior to the need for intensive care [217]. Decreased levels compared to normal controls [66].	-
Endothelin-1	Potent vasoconstrictor peptide produced by endothelial cells and degraded predominantly in the pulmonary vasculature.	Increased in the plasma of patients hospitalised with COVID-19 [227]. Auto-antibodies against endothelin-1 type A receptor have been found to be significantly increased in COVID-19 patients with an unfavourable disease course [228].	Elevated in long COVID patients compared to healthy controls [31].
sACE2	Converts angiotensin II into angiotensin (1–7).	Elevated sACE2 levels in one critically ill COVID-19 patient with ARDS [234]. Higher sACE2 levels in COVID-19 patients compared to healthy controls [235]. Lower levels in patients with suspected COVID-19 compared to the control group [238]. sACE2 is upregulated in COVID-19-induced ARDS [236].	No significant difference between long COVID patients and normal controls [31].
sACE	Hydrolyses angiotensin I to angiotensin II and breaks down bradykinin.	Baseline sACE activity in the sera of severe and non-severe COVID-19 patients was decreased compared to normal controls, with the lowest levels seen in the severe COVID-19 group [244]. sACE activity upon admission did not reflect disease severity [247]. Similar sACE activity in COVID-19-induced ARDS and non-ARDS patients [236]. sACE activity was lower in patients with COVID-19 vs. controls [245,246].	No significant difference between long COVID patients and normal controls [31].
eNOS	Expressed in a variety of cells, including endothelial cells, which constitutively produce nitric oxide (NO).	eNOS is downregulated in COVID-19-induced ARDS [236].	-
ADM	Circulating peptide hormone with vasodilatory effects. Reduces vascular (hyper) permeability and promotes endothelial stability and integrity.	ADM RNA expression is increased in patients with severe COVID-19 [259]. Non-survivors showed significantly higher ADM levels than survivors, and could predict 28-day mortality [260]. In COVID-19 patients, ADM was a good predictor of patient mortality [257]. ADM levels were associated with short-term mortality in critically ill COVID-19 patients [261].	-

The biomarkers are listed as presented in the main text. ADM = adrenomedullin; Ang-1 = angiopoietin-1; Ang-2 = angiopoietin-2; eNOS = endothelial nitric oxide synthase; EphA2 = ephrin type-A receptor 2; PAI-1 = plasminogen activator inhibitor 1; sACE = soluble angiotensin converting enzyme; sCD40L = soluble CD40 ligand; Sepcr = soluble endothelial protein C receptor; sICAM-1 = soluble intercellular adhesion molecule 1; sPECAM-1 = soluble platelet endothelial cell adhesion molecule 1; sTM = soluble thrombomodulin; sTREM-1 = soluble triggering receptor expressed on myeloid cells 1; suPAR = soluble urokinase-type plasminogen activator receptor; sVCAM-1 = soluble vascular cell adhesion molecule 1; TF = tissue factor; VE-cadherin = vascular endothelial cadherin; VEGF = vascular endothelial growth factor; VEGF-R = vascular endothelial growth factor receptor; vWf = von Willebrand factor.

## Data Availability

Not applicable.
